# Conductive Cellulose Composites with Low Percolation Threshold for 3D Printed Electronics

**DOI:** 10.1038/s41598-017-03365-w

**Published:** 2017-06-12

**Authors:** Jae Sung Park, Taeil Kim, Woo Soo Kim

**Affiliations:** Stretchable Device Laboratory, School of Mechatronic Systems Engineering, Simon Fraser University, Burnaby, BC Canada

## Abstract

We are reporting a 3D printable composite paste having strong thixotropic rheology. The composite has been designed and investigated with highly conductive silver nanowires. The optimized electrical percolation threshold from both simulation and experiment is shown from 0.7 vol. % of silver nanowires which is significantly lower than other composites using conductive nano-materials. Reliable conductivity of 1.19 × 10^2^ S/cm has been achieved from the demonstrated 3D printable composite with 1.9 vol. % loading of silver nanowires. Utilizing the high conductivity of the printable composites, 3D printing of designed battery electrode pastes is demonstrated. Rheology study shows superior printability of the electrode pastes aided by the cellulose’s strong thixotropic rheology. The designed anode, electrolyte, and cathode pastes are sequentially printed to form a three-layered lithium battery for the demonstration of a charging profile. This study opens opportunities of 3D printable conductive materials to create printed electronics with the next generation additive manufacturing process.

## Introduction

Design of 3D printable conductive composites focuses both on the investigation of network percolation with the conductive fillers and optimization of extrusion printability with paste extruder. Electrically conductive silver nanowire (AgNW) has been proposed as a conductive filler for the application of conductive nanocomposites in three dimension (3D Conductor)^1, 2^. AgNW shows metallic electrical conductivity (1.6 × 10^−6^ Ω∙cm) and capability of percolated network formation from its high aspect ratio (typically, 50~500) within a composite matrix^1, 3, 4^. I. Xu *et al*.^1^ and White *et al*.^2^ have demonstrated an AgNW based conductive nanocomposite with a maximum electrical conductivity of 10^3^ S/cm. Thanks to the promising conductivity of AgNW, the reported conductivity of AgNW based composites presents superior conductivity compared to carbon based conductive composites introduced elsewhere^5–7^. Not only the high conductivity of AgNW benefits the nanocomposite but also the high aspect ratio of AgNW provides the advantage in composite design by allowing the lower percolation thresholds. In a composite with a conductive filler inclusion, the conductive fillers can form conducting paths when they are in contact or at proximity where electrons can jump between fillers (tunneling effect)^8^. The described conductive path is called a percolation network which is essential for a composite to conduct electrons. Both the demonstrations from White *et al*. and Xu *et al*. have presented low percolation thresholds of 2.3 vol. % and 0.29 vol. % of AgNW concentrations. The reported thresholds of AgNW based composites are relatively low as the percolation threshold of a composite with silver nanoparticle (AgNP) has been found as 12 vol. %^9^ while it typically ranges between 3~25 wt. %^10, 11^ for carbon black (CB) based composites.

A sodium carboxymethyl cellulose (CMC) is often used and researched as a matrix material due to its viscosity thickening capability and thixotropic rheology^12–14^. Also, it has been drawing attentions for its water processability and proposed as a replacement material for conventional polymeric binders such as polyvinylidene fluoride (PVDF) which require the use of toxic solvents for their manufacturing^15–17^. Merging conductive AgNW filler with the CMC matrix can create a 3D Conductor.

The utilization of the printable 3D Conductor can be extended to direct 3D printing of electronics^18^. A lithium battery has a structure of stacked composite layers and has been reported as one of the paste extrusion based 3D printed electronic components. A recent research from Sun *et al*.^19^ and Fu *et al*.^20^ have demonstrated 3D printed lithium batteries made of cathode (lithium iron phosphate, LFP) and anode (lithium titanate, LTO) composite pastes. The shear thinning property of the composite paste is verified with the viscosity study which enabled layer stacking of the battery pastes. Especially, Fu *et al*.^20^ has added conductive reduced graphene oxide (rGO) particles to the paste to enhance the conductivity of electrodes. Because of a highly conductive rGO loading (30 wt. %) in the paste, the resulting cathode and anode electrodes have respectively displayed resistivity of 3 × 10^−2^ Ω·cm and 1.6 × 10^−1^ Ω·cm which is about 5^th^ order of magnitude less than the pastes from the research conducted by K. Sun *et al*.^19^. The enhanced conductivity in the electrodes results in significant reduction of internal resistance of the battery, which promotes the electrochemical performance of the battery^20^. The recent research activities suggest the practical use of the highly viscous and shear thinning pastes to create the printed electronics with the next generation additive manufacturing process.

**Figure 1:**
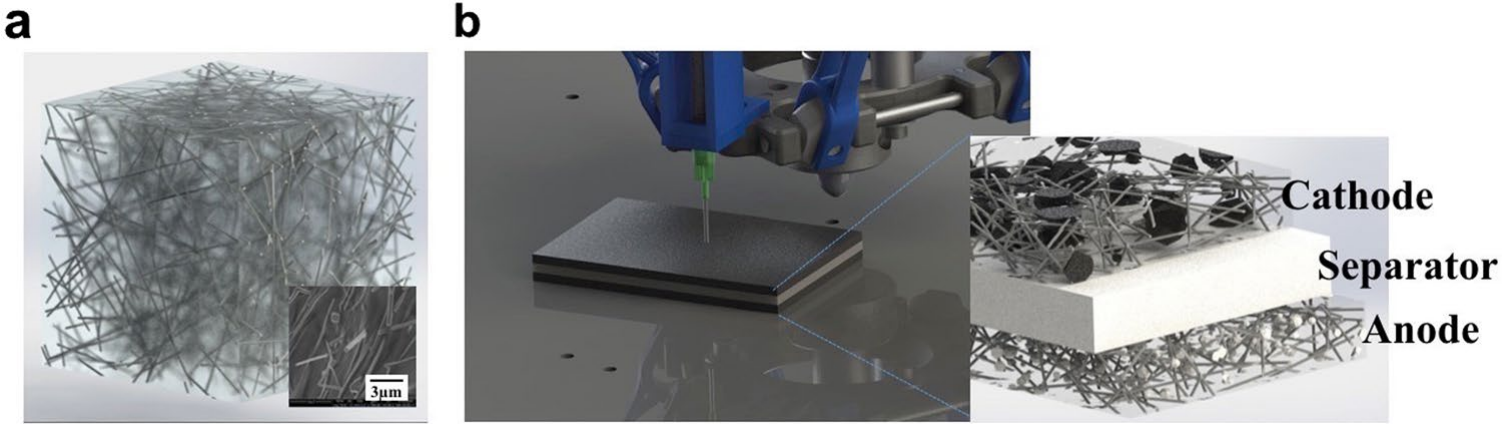
Schematic designs of cellulose composite and 3D printing of battery composites. (**a**) Computer aided design (CAD) model of AgNW/CMC 3D Conductor. Inset shows the scanning electron microscopy (SEM) image of the AgNW/CMC 3D Conductor, (**b**) CAD representation of three-layered 3D printed battery.

Figure 1a shows an AgNW based printable 3D Conductor. To the best of our knowledge, no report has demonstrated percolation studies of AgNW within CMC matrix with high aspect ratio about 200. Thus, we aim to establish a percolation threshold of the 3D Conductor with lower concentration of AgNW as high aspect ratio fillers. And it will be investigated by computational simulations as well as experimental evaluations. Several computer-aided design (CAD) models are designed with various filler fractions to simulate electrical conductivity of the 3D Conductors. Also, for the experiments on AgNW/CMC 3D Conductor, the composite samples are prepared with various filler fractions (from 0.3 vol. % to 1.9 vol. %) and their electrical resistivity values are measured to find a percolation threshold.

## Results and Discussion

The schematic of 3D printable battery using designed 3D conductor composite is described in Figure 1b. Cathode and anode materials are designed by combining cathode and anode active materials with the designed 3D Conductor. Rheology study of the paste is carried out to observe thixotropism (shear thinning property) which is essential for 3D printable paste^20, 21^. Also, a performance demonstration from the fabricated 3D printed battery is introduced.

**Figure 2:**
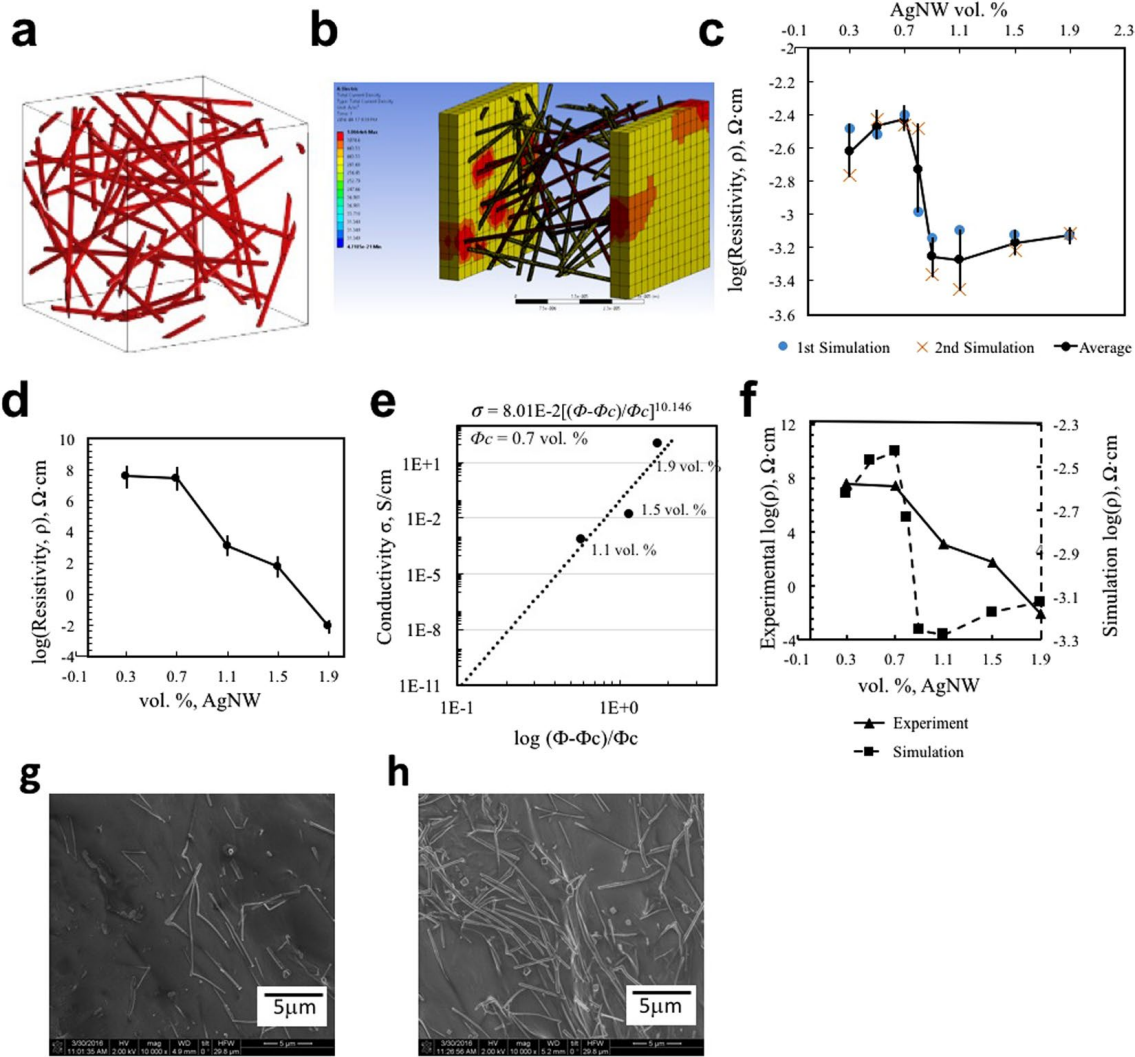
Computational Design and Percolation Evaluation of 3D Conductor and Experimental Percolation Threshold of 3D Conductor (**a**) Generated geometry of AgNW/CMC 3D Conductor by Digimat, (**b**) Image of computational simulation with ANSYS Workbench. Red colored area represents higher current density, (**c**) Simulation result of resistivity change with different AgNW concentrations. (**d**) Resistivity plot of the 3D Conductor. (**e**) Power law plot behavior of the 3D Conductor. (**f**) Comparative plot of electrical resistivity from experiment and simulation with different AgNW concentrations. (**g**) SEM image of 0.3 Vol. % AgNW in CMC, (**h**) SEM image of 1.9 Vol. % AgNW in CMC.

The electrical percolation threshold is firstly investigated by computational simulation with the AgNW filled CMC based 3D Conductor. The simulation is performed by measuring resistivity of the composite models of eight different AgNW concentrations ranging from 0.3 vol. % to 1.9 vol. %. For each concentration, two different AgNW/CMC geometries are generated and the average resistivity values between the 1^st^ and 2^nd^ simulations are used to find the percolation threshold. As shown in Figure 2a, the computational simulation for electrical conductivity started with designing an AgNW/CMC composite with a composite modeling software (Digimat-FE, e-Xstream engineering). The Digimat allows a user to create 3D Representative Volume Element (RVE) composed of a matrix phase filled with various filler material phases automatically. We created different AgNW network structures using Digimat by changing filler volume fractions only. The different results from randomly generated 1^st^ and 2^nd^ simulation come from the changed distribution of AgNWs in each sample of AgNW/CMC 3D Conductor for two cases. This change of AgNW arrangement is randomly input by Digimat. To model our AgNW/CMC 3D Conductor, random oriented cylindrical filler is set with an aspect ratio of 200. Because the network connectivity is determined by the dimension of AgNWs such as aspect ratio, we used the fixed aspect ratio of 200 for all simulation like the one in the experiment. If higher aspect ratio of AgNW is added in the composite, the percolation threshold is significantly reduced^2^. Network connectivity was not directly quantified in this model of simulation by Digimat. Instead, we used the resistivity as an indicator through additional electrical conduction simulation by ANSYS. The voltage change of the electrical conduction simulation in ANSYS showed the change of network connectivity indirectly depending on the change of filler volume fraction.

In order to investigate a percolation threshold of the 3D Conductor, its model is designed with two thin copper electrodes added to two sides of the composite model to apply electrical conduction in simulation. The electrically conductive characterization is carried out by ANSYS Workbench (ANSYS, Inc.). In ANSYS Workbench, electrical resistivity is set to 1.58 × 10^−6^ Ω∙cm for AgNW^4^, 1.015 × 10^11^ Ω∙cm for CMC^22^, and 1.68 × 10^−6^ Ω∙cm for copper^23^ respectively. Then a voltage change across the composite is measured while grounding 2^nd^ copper electrode and passing a current through the composite model. A resistance *R* of the composite model is computed with a simple Ohm’s law (equation 1) and resistivity (*ρ*) is then calculated for the composite (equation 2).

1 $$R=\frac{V}{I}$$

2 $$\rho =R\cdot \frac{Cross\,Sectional\,Area\,of\,RVE}{Length\,of\,RVE}$$

where V is the voltage change across the composite and I is the applied current.

Figure 2b depicts current density distribution over the composite model (CMC matrix’s visibility hidden for better understanding). The current flowing through the percolation of AgNW network is observed. Moreover, higher number of percolations and electrical couplings between 1^st^ and 2^nd^ electrodes have been observed with increased AgNW concentration.

Figure 2c shows a clear trend of decreased resistivity by increasing concentration of AgNW. The average resistivity at 0.7 vol. % is 3.75 × 10^−3^ Ω∙cm. With a slight increase of AgNW concentration, the resistivity drops to 5.73 × 10^−4^ Ω∙cm at 0.9 vol. %. The significant resistivity drop starts at 0.7 vol. % indicates that the 3D Conductor possesses a percolation threshold *Φ*_*c*_ of 0.7 vol. %. The variability of resistivity for 1^st^ and 2^nd^ simulation is closely related to different arrangements of AgNWs. The different results from randomly generated 1^st^ and 2^nd^ simulation come from the changed distribution of AgNWs in each sample of AgNW/CMC 3D Conductor for two cases by the Digimat. For the same vol. % of AgNW, the resistivity can be different because the conductive AgNW network will be different depending on the geometry. This variability is the highest especially near the AgNW threshold vol. % because the resistivity can be varied dramatically depending on the extent of percolation based on the arrangements of AgNWs in this region. However, the variability of resistivity is lower for higher AgNW vol. %. Each AgNW is positioned in a different way by randomly distributing simulation maintaining the same constant variables like volume fraction and aspect ratio of AgNWs.

Now, to find out the percolation threshold *Φ*_*c*_ experimentally, AgNW/CMC 3D Conductor paste is prepared with five different AgNW filler contents ranging from 0.3 vol. % to 1.9 vol. %. The AgNW and CMC volume fractions of each sample are calculated using density and weight fraction values of AgNW and CMC particles as shown in following equations.

3 $${\Phi }_{AgNW}=\frac{\frac{{W}_{f,AgNW}}{{\rho }_{AgNW}}\,}{\frac{{W}_{f,AgNW}}{{\rho }_{AgNW}}+\frac{{W}_{f,CMC}}{{\rho }_{CMC}}}$$

4 $${\Phi }_{CMC}=\frac{\frac{{W}_{f,CMC}}{{\rho }_{CMC}}\,}{\frac{{W}_{f,AgNW}}{{\rho }_{AgNW}}+\frac{{W}_{f,CMC}}{{\rho }_{CMC}}}$$

where ${\Phi }_{AgNW}$, ${\Phi }_{CMC}$, ${W}_{f,AgNW}$, ${W}_{f,CMC}$, ${\rho }_{AgNW}$, and ${\rho }_{CMC}$ are AgNW volume fraction, CMC volume fraction, AgNW weight fraction, CMC weight fraction, AgNW density (10.6 g/cm^3^)^2^, and CMC density (1.6 g/cm^3^)^24^ respectively. A complete list of samples with their content loading and resistivity is summarized in Table 1.

**Table 1:** Prepared AgNW/CMC composite with different concentrations and their resistivity.

Samples	CMC (vol. %)	AgNW (vol. %)	Solid Content (wt. %)	Resistivity (Ω∙cm)
#1	99.7	0.3	25	3.74 × 10^7^
#2	99.3	0.7	25	2.57 × 10^7^
#3	98.9	1.1	25	1.25 × 10^3^
#4	98.5	1.5	25	5.92 × 10^1^
#5	98.1	1.9	25	8.38 × 10^−3^

3D conductor composite is prepared by addition of AgNW and CMC in deionized water. And solid content of the paste $(({\rm{M}}{\rm{a}}{\rm{s}}{\rm{s}}\,{\rm{o}}{\rm{f}}\,{\rm{A}}{\rm{g}}{\rm{N}}{\rm{W}}+{\rm{M}}{\rm{a}}{\rm{s}}{\rm{s}}\,{\rm{o}}{\rm{f}}\,{\rm{C}}{\rm{M}}{\rm{C}})/\,{\rm{M}}{\rm{a}}{\rm{s}}{\rm{s}}\,{\rm{o}}{\rm{f}}\,{\rm{P}}{\rm{a}}{\rm{s}}{\rm{t}}{\rm{e}})$ is set to 25 wt. % in order to create high viscosity for the best 3D printability and layer stacking ability. Five specimens from each concentration of AgNWs, 25 specimens in total, are prepared. As shown in Figure 2d, the percolation threshold Φc is found to be 0.7 vol. % and a significant resistivity reduction about 10^th^ order of magnitude (from 3.74 × 10^7^ Ω∙cm @ 0.3 vol. % to 8.38 × 10^−3^ Ω∙cm @ 1.9 vol. %) has been observed. The standard error of resistivity for each formulation is given in Table 2. Data from five specimens were used to calculate the standard error of resistivity of each concentration of AgNWs.

**Table 2:** Standard Error of Resistivity.

Vol. % AgNW	0.3	0.7	1.1	1.5	1.9
Standard Error of Resistivity (ohm*cm), rho	1.49*10^7^	1.15*10^7^	6.26*10^2^	1.80*10^1^	4.52*10^−4^

In a composite including conductor and insulator phases, the electrical conduction behavior can be approximated by a power law^25^. The power law is mathematically represented by an equation shown below^2^.

5 $$\sigma \approx {[\frac{({\rm{\Phi }}-{\rm{\Phi }}c)}{{\rm{\Phi }}c}]}^{\alpha }$$

where σ is electrical conductivity, *Φ*_*c*_ is percolation threshold, *Φ* is filler volume fraction, and *α* is critical exponent. When the critical exponent *α* is between 1.1 and 1.3 the percolation network is known to be two dimensional (2D) percolation, and the α is in the range of 1.6 to 2 in case of a network of three dimensional (3D) percolation^26^. Figure 2e shows a power law plot of the AgNW/CMC 3D Conductor. The percolation threshold *Φ*_*c*_ (0.7 vol. %) with conductivity values of the higher concentration (1.1, 1.5, and 1.9 vol. %) of AgNWs are utilized to find critical exponent of the 3D Conductor. From the best fit straight line with R^2^ value of 0.83, the critical exponent is found to be 10.146. This indicates that the tunneling effect is a dominant charge transport mechanism in the AgNW/CMC 3D Conductor^27^. Tunneling effect is usually a main conductivity mechanism of composites made of conductive fillers embedded in insulative matrix^28^. Due to the thin insulative layer surrounding the conductive fillers, the fillers conduct electrons by tunneling effect rather than through their mechanical contacts. It has been reported that the higher volume concentration of the conductive fillers decreases the electron tunneling distance between the fillers^27^. And the conductive fillers neighboring within the tunneling distance form “clusters”. The connections of clusters then form the percolation network enabling electric current to flow through the composite^27^. SEM images in Figure 2g and h show conductive fillers embedded in an insulative polymer matrix. Figure 2h with higher volume fraction of fillers shows the decreased gap between fillers compared to the fillers’ gap in Figure 2g with lower volume fraction. Figure 2h also shows the clusters which form the localized percolation networks. This is matched well with the resistivity change result in the simulation. The resistivity from the simulation result was higher for the case of 0.3 vol. % (Figure 2g) and was dramatically decreased for the case of 1.9 vol. % (Figure 2h).

The experimental resistivity data of the AgNW/CMC composite has demonstrated a good agreement with the simulation results. For comparison, a plot of merged experimental and simulational resistivity results is shown in Figure 2f. The lower level of resistivity from the simulation compared to the experimental result may be ascribed to an ideal overlapping of the AgNWs (inter-penetrable AgNWs), in contrast to the real AgNW network where individual nanowire has thin polyvinylpyrrolidone (PVP) coating which causes imperfect AgNW fusion after annealing^29^. The low percolation threshold of 0.7 vol. % and the superior electrical conductivity (8.38 × 10^−3^ Ω∙cm) compared to other kinds of AgNW composite^2, 30^ suggest an excellent harmony of AgNW filler and CMC matrix for generating a conductive 3D Conductor.

For a fabrication of lithium battery electrode pastes, lithium iron phosphate (LFP, <5 um particle size, Sigma Aldrich) and lithium titanate (LTO, <200 nm particle size, Sigma Aldrich) active materials are respectively chosen for cathode and anode. LFP and LTO suffer inferior electrical conductivity. The use of these materials has been challenging for their low electrical conductivity ~10^−9^ S/cm for LFP and ~10^−13^ S/cm for LTO respectively^31, 32^. Substantial efforts are made to increase the conductivity of LFP and LTO by cationic doping^33, 34^, carbon coating^35^, mixture with conductive nanocarbon in cathode^33, 34^, or size reduction of LTO particle to reduce diffusion length of Li + ion and electrons^36^. In this research, we investigate fabrication of conductive LFP and LTO electrodes facilitated with addition of highly conductive AgNW filler. 3D printable cathode and anode pastes are fabricated with composition shown in Table 3.

**Table 3:** Prepared cathode and anode composite paste.

	Active Material (vol. %)	CMC (vol. %)	AgNW (vol. %)	Solid Particles (wt. %)
Cathode	78.1	20	1.9	40
Anode	78.1	20	1.9	40

**Figure 3:**
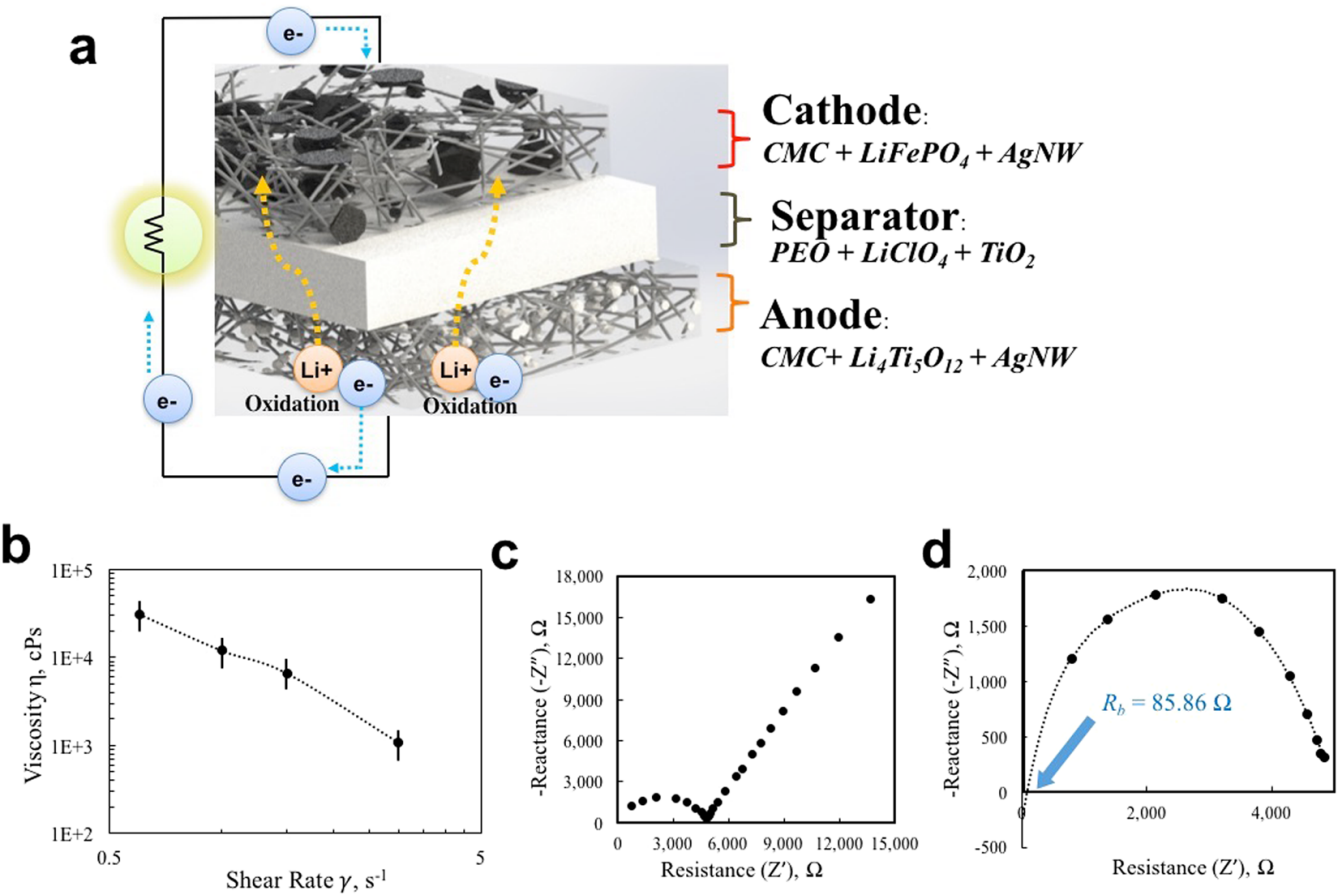
Design of Printable Pastes for Battery Components and Ionic Conductivity of Printable Electrolytes. (**a**) Schematic overview of 3D printed battery. Larger black particles represent LFP particles while smaller white particles represent LTO particles, (**b**) Viscosity change of anode paste with increasing shear rate. The anode paste displays strong shear thinning behavior which benefits the 3D printability. Nyquist plot of PEO-LiClO_4_-TiO_2_ electrolyte, (**c**) Impedance of the electrolyte with different frequency values from 20 Hz to 2 MHz, (**d**) Zoomed in plot at high frequency region. Dotted trend line is drawn to approximate the bulk resistance *R*_*b*_ of the electrolyte.

As illustrated in Figure 3a, pastes are carefully designed to acquire 3D printability (high viscosity by CMC), high electrical conductivity (by AgNW), and high lithium active material loadings. The electrode pastes are designed to include high concentration of the solid particles (active material, CMC and AgNW) of 40 wt. % to minimize possible volume shrinkage of printed paste upon drying. For the vol. % calculation, densities of 3.6 g/cm^3^ for LFP particle^37^ and 3.539 g/cm^3^ for LTO^19^ are used.

The fabricated shows electrical conductivity of 4.21 × 10^−3^ S/cm and 1.64 × 10^−4^ S/cm for cathode and anode paste respectively which are several orders of magnitude higher conductive values than that of the pure LFP and LTO^31, 38^. Also, the anode and cathode pastes show conductivity levels higher than conductivity of reported battery electrode pastes which are 4.35 × 10^−4^ S/cm and 4.76 × 10^−6^ S/cm for LFP-based and LTO-based electrode pastes respectively^39^. This indicates that even the low 1.9 vol. % AgNW concentration can effectively enhance the conductivity of electrodes. However, these conductivities are lower than the conductivity of 1.9 vol. % AgNW concentration AgNW/CMC sample which is 1.19 × 10^2^ S/cm. We believe that the large solid LFP and LTO particles within the electrode paste matrix are interfering an even distribution of the AgNW and discouraging the percolation network formation in the matrix.

Electrode pastes must have a superior 3D printability, in other words, a capability to be stacked layer by layer without layer bucklings. Rheology of the anode paste (LTO/AgNW/CMC) has been studied by measuring viscosity at different shear rates with a viscometer (microVISC™, RheoSense). As can be seen from Figure 3b, the anode paste demonstrated a strong shear thinning behavior. By increasing shear rate from 0.6 s^−1^ to 3 s^−1^, viscosity of the paste drops from 30,832 cps (viscosity level of hot fudge) to 1,063 cps (viscosity level of detergent)^40^.

Shear rate at the extrusion nozzle can be calculated by using following equation^37^.

6 $${\rm{\gamma }}=\frac{4\cdot Q}{\pi \cdot {{R}_{n}}^{3}}$$

where γ is shear rate (s^−1^) in the nozzle, *Q* is extrusion flow rate (0.0002 ml/s or 0.2 mm^3^/s), and *R*_*n*_ is inner radius of nozzle (0.42 mm). The calculated shear rate for extruding LTO paste is 1.72 s^−1^. This indicates that the viscosity of the paste is around 6,000 cps during extrusion through the 0.84 mm-sized nozzle.

A quantitative analysis on the rheology of the anode paste is also done with Ostwald-de-Waele equation (Power Law) as shown in below equation^13^.

7 $$\tau =\kappa {(\gamma )}^{n}$$

where *τ* is shear stress (Pa), κ is consistency index (Pa s^n^), *γ* is shear rate (s^−1^), and *n* is flow behavior index. By using this formula, we have found the flow index *n* to be 0.2745 which is close to 0, meaning that the anode paste demonstrates a strong shear thinning rheology^13^. This implies that the significant decrease of viscosity at higher shear rate allows easier extrusion through narrow printing nozzles while the raised viscosity after extrusion lets the paste to maintain its filamentary shape. Unfortunately for cathode paste, viscosity measurement is unavailable due to large size of LFP particles which may block a paste flow channel of the viscometer. However, the fabricated cathode paste also has shown practical extrusion capability and maintains filamentary shape after extrusion.

For the electrolyte layer of battery, PEO-based electrolyte is chosen. LiClO_4_ (battery grade, Sigma Aldrich) lithium salt and TiO_2_ (<25 nm particle size, Sigma Aldrich) ceramic filler are added to PEO to enhance ionic conductivity^41^. PEO is a high molecular weight polymer (M_w_~900,000, Sigma Aldrich) which is able to create high viscosity upon a dissolution by acetonitrile solvent (Sigma Aldrich). For the electrolyte composition, [EO]/[Li] molar ratio of 8 and TiO_2_ load of 10 wt. % is used^41–43^.

As shown in Figure 3c, a Nyquist plot is plotted by sweeping frequency from 20 Hz to 2 MHz by an LCR meter (E4980A High Precision LCR Meter, Agilent Technologies) to find ionic conductivity of the electrolyte. As expected, a Nyquist plot in a shape of slanted line at lower frequency followed by a large semicircle at high frequency has been observed^44, 45^. The impedance characteristic of PEO-based electrolyte comes from the combined effect by a capacitance of stainless steel electrode in series with a parallel combination of bulk ionic resistance of the electrolyte and electrolyte capacitance^46^. At high frequency, an intersection of the semicircle and the real axis (Z’) gives a bulk resistance of the electrolyte^46^. By extending a trend line of the semicircle to intercept real axis, we can approximate bulk resistance of the electrolyte as 85.86 Ω (Figure 3d).

Ionic conductivity can be calculated using a following equation with the bulk resistance of the electrolyte^47, 48^.

8 $$\sigma =\frac{t}{A\times {R}_{b}}\,S\,c{m}^{-1}=1.83\,\times {10}^{-4}S\,c{m}^{-1}$$

where *t* is a thickness of the electrolyte (0.24 mm), *A* is a cross sectional area of the sandwiching stainless disk (1.53 mm^2^), and *R*_*b*_ is a bulk resistance of the electrolyte (85.86 Ω). The demonstrated ionic conductivity of our electrolyte falls in a useful ionic conductivity level and agrees with other established reports based on PEO-LiClO_4_ electrolyte^41, 44, 49^.

**Figure 4:**
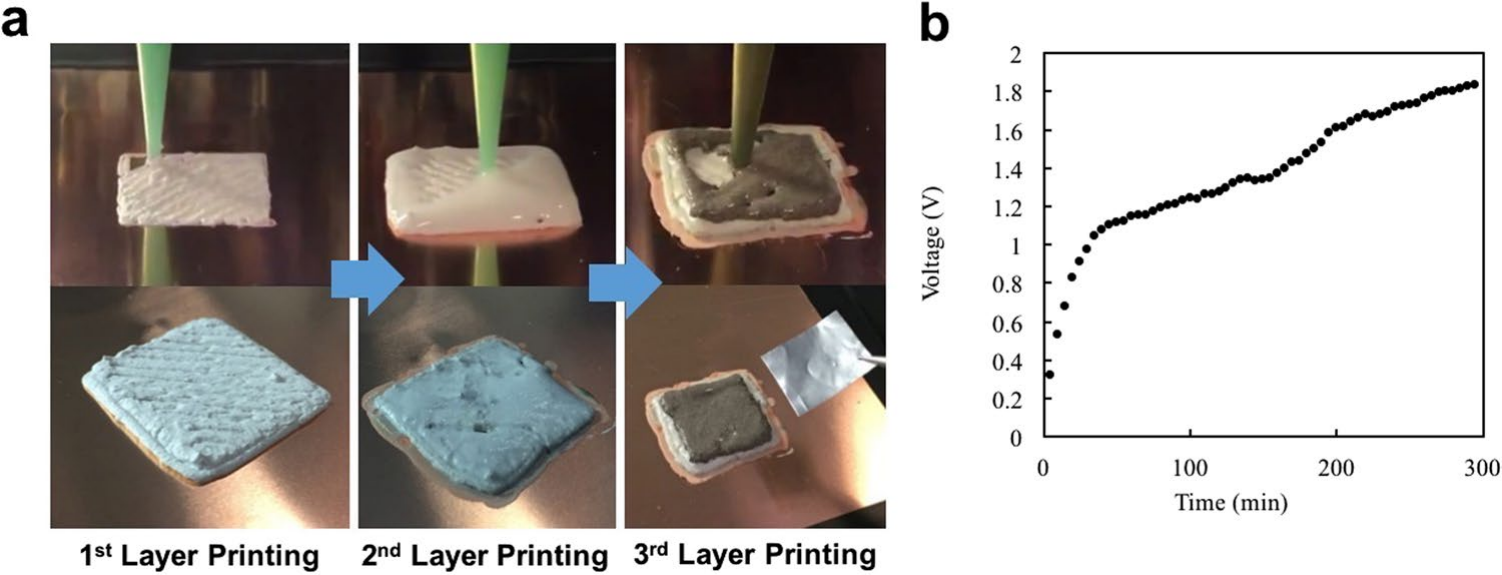
(**a**) Images of 3D printing process of the 3D printed battery. Anode, electrolyte and cathode are sequentially printed to form a three-layered battery structure and Performance of 3D Printed Battery (**b**) Plot of battery’s charging profile. Voltage measurement of the battery.

Our 3D printed battery has a three-layer-stacked structure, in which, first layer is anode, and second layer is electrolyte followed by a third layer of cathode. Each layer is square shaped with a size of 2 × 2 cm^2^. The anode paste is first printed on a copper current collector. A 0.84 mm nozzle is used to print the anode paste with a slow movement with print head speed of 0.7 mm/s and extrusion rate of 0.0002 ml/s. For the printing of viscous electrolyte solution, the same printing parameter is used. And a single layer of the electrolyte is printed. The printed sample has been vacuum dried at 60 °C for 24 hours to remove possible moisture from the anode and electrolyte layers. On the other hand, a larger 1.54 mm nozzle, 1.2 mm/s of printing head movement speed and extrusion rate of 0.00083 ml/s are used to print the cathode paste. On top of the non-dried cathode layer after extrusion, an aluminum sheet is placed as a current collector for cathode. The complete three-layered battery sample has been dried in vacuum oven at 60 °C for 24 hours for solvent removal. Figure 4a demonstrates a 3D printing process of the battery.

Constant voltage (CV) charging has been performed to see charging characteristic of the battery. Supply voltage of 2 V is applied to the battery and cell voltage is measured every 5 minutes with CT2001a battery tester (Landt Instruments). The voltage profile during charging is shown in Figure 4b. Discharged state of the battery shows an open circuit voltage of 0.32 V and it increases to about 1.2 V after 1 h 25 min and around 1.8 V after 4 h 35 min. The voltage of a cell is determined by the potential difference between the cathode and anode. Potential for LFP range between 3.2 V and 3.5 V while for LFP it is about 1.55 V which leads to a potential of LFP/LTO full cell to be around 1.8 V^19, 50, 51^. Demonstrated cell voltage of our 3D printed battery agrees with reported cell voltages of LFP/LTO batteries which are in the range of 1.5~1.8 V^52^.

## Conclusion

In this research, a printable 3D Conductor having strong thixotropic rheology has been designed and investigated with highly conductive AgNW fillers. The optimized electrical percolation threshold from both simulation and experiment is started from 0.7 vol. % of AgNW which is significantly lower than other composites using low aspect ratio conductive fillers. High conductivity of 1.19 × 10^2^ S/cm has been achieved from the AgNW/CMC 3D Conductor with 1.9 vol. % loading of AgNW. Utilizing the high conductivity of the AgNW/CMC 3D Conductor, battery electrode pastes are designed and demonstrated for the 3D printing process. Rheology study shows superior printability of the electrode pastes aided by the CMC’s strong thixotropic rheology. The designed anode, electrolyte, and cathode pastes are sequentially printed to form a three-layered lithium battery for the demonstration of a charging profile. The study on the AgNW/CMC 3D Conductor paste opens opportunities of 3D printable conductive pastes to create printed electronics with the next generation additive manufacturing process.

## Materials and Methods

### Preparation of AgNW/CMC 3D Conductor

AgNW is synthesized with typical polyol process to create uniform and high aspect ratio AgNWs^4^. AgNWs with a diameter of 100~150 nm and a length about 20 μm (aspect ratio of 133~200) is achieved^53^. Then a solution of AgNW is prepared by dispersing AgNW in deionized (DI) water with an ultrasonicator for 1 minutes (Ultrasonic Bath model 2510, Branson®). Prepared AgNW ink and CMC powder (M_w_ ~250,000, degree of substitution 0.7, Sigma Aldrich) is thoroughly mixed in preset volume fractions. For percolation threshold study of the AgNW/CMC nanocomposite paste, five composite samples are fabricated with different AgNW concentrations of 0.3, 0.7, 1.1, 1.5 and 1.9 vol. %. Applying high temperature (>150 °C) has been reported to weld the junctions of AgNWs in contact in turn increasing the conductivity of the percolation network^54, 55^. Thus, the prepared AgNW/CMC composite samples are thermally annealed in a hot oven at 160 °C for 50 minutes.

### Computational simulation of electrical conduction

Models of AgNW/CMC 3D Conductor is generated with a composite modeling software (Digimat-FE, e-Xstream engineering). After the composite model generation by Digimat, the individual matrix and filler geometry files are exported in.step (.stp) format and a CAD software Autodesk Inventor (Autodesk Inc.) is used to merge AgNW and CMC matrix. Then two thin copper electrodes are added at two ends of the merged AgNW/CMC model. For electrical conduction characterization, a simulation software ANSYS Workbench (ANSYS, Inc.) is used. The 2^nd^ copper plate is grounded and a current of 1 μA is applied to the 1^st^ copper plate and voltage drop across the composite has been measured.

### Preparation of electrode pastes

To fabricate 5 grams of cathode paste (40 wt. % solids), AgNW (0.121 g) is dispersed in 3 g of DI water followed by a short (1 min) ultrasonication with an ultrasonicator (Ultrasonic Bath model 2510, Branson ®). And CMC (0.192 g) powder is added and has been stirred until complete dissolve of CMC to form a viscous solution. Cathode active material, 1.687 g of LFP (<5 μm particle size, Sigma Aldrich) particles are then added to the viscous AgNW/CMC mixture and has been stirred for 20 min. Similarly, for fabrication of 5 g of anode paste (40 wt. % solids), AgNW (0.123 g), CMC (0.195 g), and 1.683 g of LTO (<200 nm particle size, Sigma Aldrich) are dispersed in 3 g of DI water with the same procedure as the LFP paste.

### Preparation and analysis of PEO-based electrolyte

For the electrolyte composition, [EO]/[Li] molar ratio of 8 and TiO_2_ load of 10 wt. % are used ^41–43^. A solvent acetonitrile (Sigma Aldrich) is used for particle dispersion. LiClO_4_ (battery grade, Sigma Aldrich) lithium salt and TiO_2_ (<25 nm particle size, Sigma Aldrich) ceramic filler are first dispersed by 300 RPM stirring in acetonitrile for 30 min while maintaining heating temperature at 60 °C. Then relative amount of PEO (Mv~900,000, Sigma Aldrich) particles are added to the solution and kept stirred for 3 hours until full dissolution of PEO and viscous liquid formation. To carry out impedance analysis on the fabricated electrolyte, the sticky electrolyte is poured on a stainless disk and vacuum dried at 60 °C for 24 hours. The resulting electrolyte membrane with 0.24 mm thickness is sandwiched with another stainless disk. Impedance analysis has been performed by measuring impedances at sweeping frequency from 20 Hz to 2 MHz by an LCR meter (E4980A High Precision LCR Meter, Agilent Technologies).

### 3D Printing of electrode pastes

A paste 3D printing apparatus is fabricated by integrating a commercially available delta FDM 3D printer (Orion, SeeMeCNC) and a paste extrusion system (Discovery, Structured Printing). Also, a printing head of the Orion is modified to hold a paste extrusion syringe. The original Discovery paste extruder uses a syringe pump to directly extrude paste materials through an extrusion nozzle. This leads to an undesired material wastage (up to 3 mL) on the long tubing between the pumping and the extrusion syringe. To avoid the material wastage, a hydraulic pumping mechanism is adopted to our paste extrusion system. The hydraulic pumping has two syringes; a 60 mL pumping syringe receiving primary pressure from the Discovery motor at one end while another 5 mL extrusion syringe located at the printing head for material extrusion. These two syringes are connected with the tubing which is filled with water. For anode paste extrusion, a 0.84 mm nozzle with print head movement speed of 0.7 mm/s and extrusion rate of 0.0002 ml/s are used. 0.8 mm thick anode layer is acquired after 15 min drying on a 70 °C heated printing bed. Then an electrolyte layer is printed by using same printing parameter with anode paste. Vacuum drying at 60 °C for 24 hours has been applied to the printed sample to remove possible moisture. Lastly, a cathode layer is printed through a 1.54 mm nozzle with extrusion rate of 0.00083 ml/s and printing movement speed of 1.2 mm/s. Then the complete printed sample has been again vacuum dried at 60 °C for 24 hours^56–65^.
